# Understanding Macroalgae: A Comprehensive Exploration of Nutraceutical, Pharmaceutical, and Omics Dimensions

**DOI:** 10.3390/plants13010113

**Published:** 2023-12-31

**Authors:** Sivakumar Adarshan, Vairavel Sivaranjani Sivani Sree, Pandiyan Muthuramalingam, Krishnanjana S Nambiar, Murugan Sevanan, Lakkakula Satish, Baskar Venkidasamy, Peerzada Gh Jeelani, Hyunsuk Shin

**Affiliations:** 1 Department of Biotechnology, Alagappa University, Karaikudi 630003, Tamil Nadu, India; sadarshan1999@gmail.com; 2 Department of Biotechnology, Karunya Institute of Technology and Sciences, Coimbatore 641114, Tamil Nadu, India; sivanisree@karunya.edu.in (V.S.S.S.); krishnasreenambiar@gmail.com (K.S.N.); micromurugans@gmail.com (M.S.); 3 Division of Horticultural Science, College of Agriculture and Life Sciences, Gyeongsang National University, Jinju 52725, Republic of Korea; shinpomo@gnu.ac.kr; 4 Department of Oral and Maxillofacial Surgery, Saveetha Institute of Medical and Technical Sciences (SIMATS), Saveetha Dental College and Hospitals, Saveetha University, Chennai 600077, Tamil Nadu, India; baskarbt07@gmail.com; 5 Applied Phycology and Biotechnology Division, Marine Algal Research Station, CSIR—Central Salt and Marine Chemicals Research Institute, Mandapam 623519, Tamil Nadu, India; pandu.pine@gmail.com; 6 Department of Biotechnology, Microbiology & Bioinformatics, National College Trichy, Tiruchirapalli 620001, Tamil Nadu, India; mejeelani@gmail.com

**Keywords:** macroalgae, natural products, nutraceuticals, omics approaches, pharmaceuticals

## Abstract

Driven by a surge in global interest in natural products, macroalgae or seaweed, has emerged as a prime source for nutraceuticals and pharmaceutical applications. Characterized by remarkable genetic diversity and a crucial role in marine ecosystems, these organisms offer not only substantial nutritional value in proteins, fibers, vitamins, and minerals, but also a diverse array of bioactive molecules with promising pharmaceutical properties. Furthermore, macroalgae produce approximately 80% of the oxygen in the atmosphere, highlighting their ecological significance. The unique combination of nutritional and bioactive attributes positions macroalgae as an ideal resource for food and medicine in various regions worldwide. This comprehensive review consolidates the latest advancements in the field, elucidating the potential applications of macroalgae in developing nutraceuticals and therapeutics. The review emphasizes the pivotal role of omics approaches in deepening our understanding of macroalgae’s physiological and molecular characteristics. By highlighting the importance of omics, this review also advocates for continued exploration and utilization of these extraordinary marine organisms in diverse domains, including drug discovery, functional foods, and other industrial applications. The multifaceted potential of macroalgae warrants further research and development to unlock their full benefits and contribute to advancing global health and sustainable industries.

## 1. Introduction

The world is witnessing a surge in the popularity of natural products (NPs) due to their incredible therapeutic potential and positive impact on human health. Notably, around 40% of FDA-approved drugs between 1981 and 2014 were derived from NPs [1]. The marine ecosystem has piqued the interest of researchers as a potential source of NPs for various industrial applications, including food, pharmaceuticals, and cosmetics. Among the marine species, algae occupy a prominent position [2,3,4]. Algae, the primary producers of the marine ecosystem [5], have been under study for more than half a century. However, the emergence of new diseases and global malnutrition has made the scrutiny of these tiny marine species even more critical. Algae are being investigated as a potential source of various products such as antibiotics [6], proteins [7], metabolites [8], antioxidants [9], and dietary supplements [10]. Some species of algae can be mass cultivated using photobioreactors, and their short generation time reduces the overexploitation of marine resources [11].

Algae are genetically diverse and can be classified into macroalgae and microalgae [12]. Macroalgae, also known as seaweed, contain various nutrients essential for the human diet, including proteins, fibers, vitamins, fats, and minerals [13]. These macroalgae are capable of growth without relying on land areas and grow faster when compared to terrestrial plants [14]. Similar to their terrestrial counterparts, seaweeds also perform crucial ecological roles, such as serving as bioindicators of water quality and participating in bioaccumulation and bioremediation processes, thereby helping to maintain aquatic environments. In addition to their contributions to marine ecosystems, seaweeds are responsible for producing about 80% of the atmospheric oxygen, which is then used by land organisms for respiration [3]. Furthermore, due to their balanced nutrient composition, macroalgae are a promising food source and are currently utilized in various countries [15]. Seaweed contains a wealth of bioactive metabolites that have the potential to treat a variety of diseases [16]. Recent research has highlighted the pharmaceutical potential of seaweed as a complementary medicine as gelling and thickening agents in various industries [17]. Consequently, macroalgae have emerged as an alternative source of food and medicine in many countries [18]. China, the Philippines, and Indonesia are the largest seaweed-producing countries in the world [19].

As the exploration and utilization of macroalgae in therapeutic applications continues to grow, it is increasingly important to have a thorough understanding of their physiological and molecular characteristics. Omics approaches are an invaluable tool for achieving this goal. Rapid advances in omics technologies have ushered in a new era in biotechnology, extending beyond genomics. This review focuses on macroalgae as a potential source for nutraceutical and therapeutic applications and consolidates the latest advancements in this field using omics approaches.

## 2. Macroalgae—A Brief Overview

Macroalgae are a diverse group of multicellular marine organisms with a long history of cultivation and traditional use in different regions of the world. They are classified into three major phyla based on their photosynthetic pigments: red algae (Rhodophyta), green algae (Chlorophyta), and brown algae (Ochrophyta) [20]. Each of these phyla has unique properties and bioactive compounds. Red algae are rich in phycobiliproteins, pigments, phycolectins, mycosporine, unsaturated fatty acids, polysaccharides, minerals, and vitamins [21]. Both red and green algae are abundant sources of bromophenols, phenolic acids, and flavonoids, while brown algae are high in phlorotannin content [22]. Some properties of these three phyla are compared and tabulated in Table 1.

The most cultivated macroalgae are *Pyropia* spp., *Kappaphycus* spp., *Undaria* spp., *Gracilaria* spp., and *Eucheuma* spp. [19]. *Lonicera japonica*, *Undaria pinnatifida*, and *Hizikia fusiforme* are some of the notable commercially important seaweeds [30]. In brief, these marine organisms with diverse metabolites can be used as an alternative source for synthetic ingredients in pharmaceuticals and nutraceuticals.

### 2.1. Nutraceutical Potentials of Macroalgae

Seaweeds are an important source of high-quality proteins in the marine environment, which are essential in nutraceutical formulations [31]. Macroalgae contain various amino acids, including threonine, alanine, arginine, glutamic acid, and aspartic acid [32]. In addition, these organisms also contain micronutrients such as Manganese (Mn), Copper (Cu), Zinc (Zn), Iron (Fe), Cadmium (Cd), Lead (Pb) [33], and macronutrients such as phlorotannins, catechol, and quercetin [34]. Macroalgal species and their nutrients are listed in Table 2.

**Figure 1:**
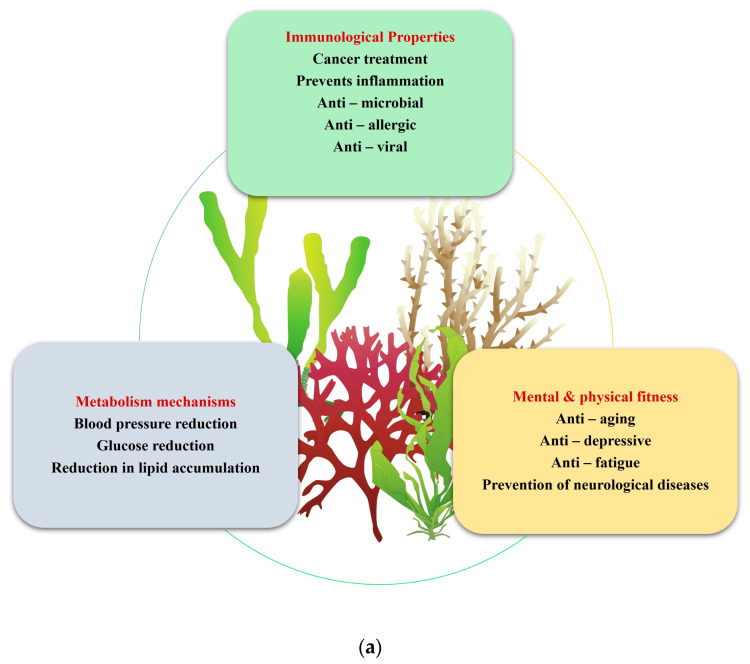

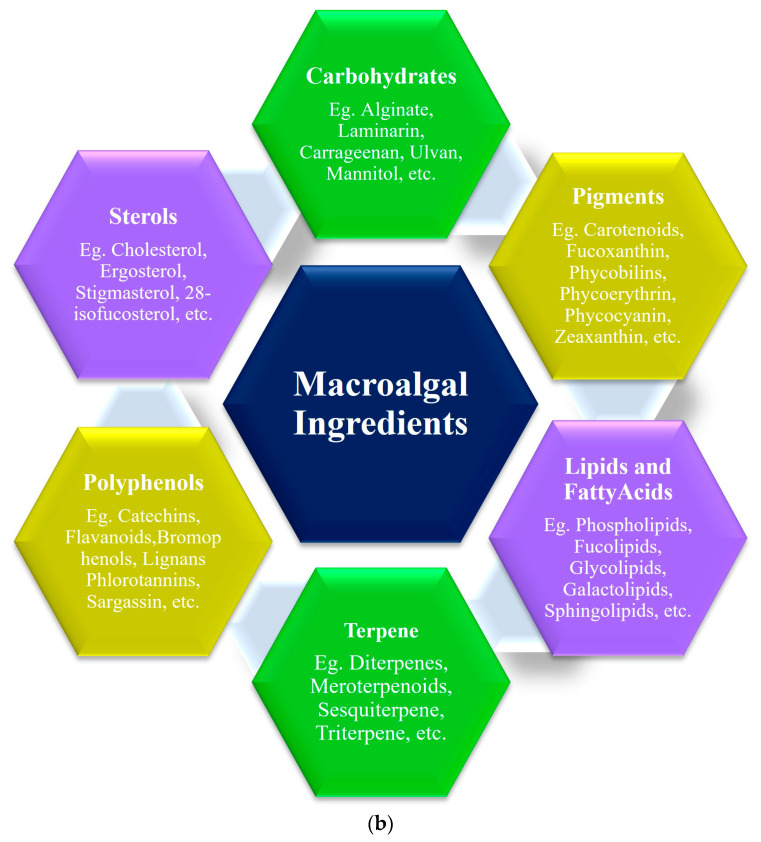
(**a**) Nutraceutical applications of macroalgae, (**b**) Macroalgal active ingredients.

A nutraceutical is a substance that has biological effects or can protect against some chronic diseases. This can be used to improve health, delay senility, increase life expectancy, or improve the body’s metabolism. The major reason macroalgae are necessary is their size, which provides an increased surface area for greater biosorption of hazardous chemicals even at low concentrations. A few nutraceutical applications are depicted in Figure 1a and the macroalgal ingredients are given in Figure 1b.

A few representative macroalgae derived compounds and their nutraceutical and therapeutic applications are given in the Table 3.

The studies show the efficiency of the nutrients derived from the marine macroalgae, which gain its importance in medical, food, cosmetics, and other industrial segments to list on.

### 2.2. Pharmaceutical Potentials of Macroalgae

Seaweeds are a rich source of bioactive compounds, particularly brown algae, which contain over 700 biochemicals with various therapeutic properties, such as anti-cancer, anti-inflammatory, antioxidant, anti-coagulant, anti-HIV, anti-diabetic, and anti-allergic activities [14]. For example, fucoxanthin, found in the brown algae *U. pinnatifida* has been shown to have significant anti-cancer, anti-inflammatory, and anti-obesity effects [104]. Fucoidan, synthesized by *Saccharina japonica*, has antiviral, anticancer, and anti-inflammatory effects [105]. *Ulva* spp. produces eicosapentaenoic and docosahexaenoic acids, which have anti-inflammatory and antioxidant activities [106]. Other important bioactive compounds present in seaweeds include phytocoerythrobilin, neoxanthin, chlorophyll-a, heparins, carrageenan, fucans, and galactans [107].

The diversity of macroalgae is a potential source of bioactive substances with biotechnological and medicinal applications. The investigation into marine natural products began in 1951 when Bergman and Feeney extracted spongothymidine and spongouridine from the sponge *Cryptotethya crypta* Laubenfels. This led to the discovery of arabinosyl cytosine (Ara-C), an anti-cancer compound with marine origins used to treat many types of leukaemia [108]. According to the existence of colors, macroalgae can be categorized into three groups: Rhodophyceae (red algae), Phaeophyceae (brown algae), and Chlorophyceae (green algae). Chlorophylls a and b are responsible for the color of Chlorophyta, while fucoxanthin, chlorophylls a, b, and c, are thought to be responsible for the greenish-brown color of Phaeophyta [109]. The pigments that give Rhodophyta its color include phycobilins such as phycoerythrin and phycocyanin. These are great sources of the vitamins A, Bl, B12, C, D, and E, riboflavin, niacin, pantothenic acid, and folic acid, as well as the minerals calcium, phosphorus, sodium, and potassium. They are also considered a source of bioactive chemicals because they can form a range of secondary metabolites [108]. In fact, macroalgae, like photosynthesizing plants, have antioxidant defense mechanisms. They also include polysaccharides and glycoproteins that contain chemicals with anti-cancer, antioxidant, anti-inflammatory, anti-HIV, anti-coagulant, anti-allergic, and anti-diabetic properties. This review focuses on the medicinal qualities of macroalgae, and the bioactive substances used to cure diseases [109].

Brown seaweed is widely used as nutritional supplements, herbal medications, and traditional treatments because they contain substantial amounts of the antioxidant fucoxanthin. Several cell culture models and animal investigations have shown that fucoxanthin has great antioxidant capacity and may be involved in controlling the Nrf2/ARE pathway. Fucoxanthin has an anti-inflammatory action, and their molecular methods of prevention are defined by the suppression of NF-B-related pathways [110]. Fucoxanthin has been shown to have anticancer properties in the MDA-MB-231 human breast cancer cell line, K562 and TK6 human leukemia cell lines, and the mice cancer model [111]. Hitoe and Shimoda, conducted a double-blind placebo-controlled trial in which capsules containing 1 and 3 mg fucoxanthin were given to moderately obese males and females for 4 weeks, resulted in decreased body weight, BMI, fat area and mass [112]. *Padina tetrastromatica* extract, which is fucoxanthin-rich, reduced the effects of high-calorie diet-induced obesity in c57bl/6j mice by inhibiting adipocytic lipogenesis, causing fat mass reduction, and lowering intracellular lipid content, adipocyte size, and adipose weight Fucoxanthin, interestingly, has anti-obesity benefits through modulating gut microbiota [112,113]. It also serves as a neurotrophic factor-like chemical that provides CNS neurons with neuroprotection and neurite outgrowth. In addition, fucoxanthin exhibits antibacterial potential and can treat disorders of the metabolic, hepatic, renal, cardiovascular, bone, ophthalmic, skin, and respiratory types [113,114]. Red and green seaweed contain the highest levels of phenolic substances, such as flavonoids, phenolic acids, and bromophenols [115]. The long-chain saturated fatty acids found in *Jania rubens*, such as n-hexadecanoic acid and docosanoic acid 1,2,3-propanetriyl ester and hexanedioic acid, as well as dioctyl ester, exhibited antimicrobial activity against 32 isolates of multidrug-resistant bacteria [116].

Red alga *Sphaerococcus coronopifolius* produces brominated cyclic diterpenes that function as antibacterial action against methicillin-resistant *Staphylococcus aureus* strains [117]. From *Kappaphycus alvarezii*, phenolic compounds, glycosides, and carbohydrates have been shown to have antibacterial effects on a variety of human pathogens [115]. Human pathogenic fungi, such as *Aspergillus* and *Candida*, showed resistance to the antifungal effects of methanolic extracts from *Corallina mediterranea*, *Hypnea musciformis*, and *Laurencia papillosa* [118]. A diterpene known as ‘Sphaerodactylomelol fraction 2′, recently discovered in *S. coronopifolius*, exhibited the most potent antifungal properties against *Candida albicans* [116]. Notably, the antifungal effectiveness of methanolic extracts from *Acanthaphora spicifra* was comparable to that of over-the-counter medications, such as ciprofloxacin and amphotericin [118]. Carrageenan from *Solieria chordalis* displayed antiviral activity toward HSV1 and had a protective influence against various sexually transmitted viruses, including genital warts and herpes simplex virus (HSV), thereby contributing to limiting the spread of HIV [119]. Additionally, the Mexican red seaweed, *S. filiformis* produces polyphenol-rich extracts with significant virucidal properties against the measles virus and prevent virus spread in vitro. For instance, residents in China have utilized crude extracts from the brown seaweed *Sargassum naozhouense* to cure fever, infections, laryngitis, and other illnesses. Vietnamese physicians have also employed species from the *Kappaphycus* and *Eucheuma* genera to lessen the likelihood of tumors, ulcers, and headaches. Furthermore, seaweed, such as sargassum, has been used to treat iodine deficient illnesses, including Goitre [120].

#### 2.2.1. Antioxidant Activity

Many scientists are actively working on natural antioxidants as safe alternatives to synthetic counterparts. As an antioxidant, R-phycoerythrin from *Palmaria palmata* and *Polysiphonia urceolata* was discovered [121]. In contrast, some synthetic antioxidants, such as butylated hydroxyl anisole (BHA), butylated hydroxyl toluene (BHT), tocopherol, and propyl gallate, have been found to cause liver damage and induce cancer [119]. Mycosporine rich seaweed has antioxidant properties. Despite having low macroalgal lipid content, it is rich in polyunsaturated fatty acids (PUFA), particularly arachidonic, eicosapentaenoic, linoleic, and octadecatetraenoic acids. These PUFAs exhibit antibacterial, antiviral, and antioxidant properties and are also known to prevent cardiovascular diseases and diabetes [122].

#### 2.2.2. Anticancer and Antiproliferative Activities

Recently, a new anticancer medication was developed using green seaweed *Cladophoropsis* sp. and red seaweed *Gracilaria foliifera* [123]. Carrageenan oligosaccharides from different red seaweed species have demonstrated anticarcinogenic activity with reduced cytotoxicity. They also exhibit synergistic effects when combined with conventional medications as well as improving the immunocompetence of cells killed by these medications [124]. Furthermore, the organic extract from the red seaweed *Rhodomela confervoides* contains 3-bromo-4,5-dihydroxybenzaldehyde bromophenols and 3-bromo-4,5-dihydroxy benzoic acid methyl ester. These compounds have shown high efficiency against the KB and A549 cancer cell lines, including Bel-7402 (associated with endocervical adenocarcinoma linked to human papillomavirus) [125].

#### 2.2.3. Anti-Inflammatory Activity

By preventing histamine release, neutrophil migration, and vascular permeability, a sulfated polysaccharide fraction from the cornea of the *Gracilaria* exhibits anti-inflammatory properties [126]. Nitric oxide (NO) production is reduced, and NF-B activation in mouse macrophages of RAW264.7 cells is hampered by Porphyrans derived from *Porphyra* sp., which exhibits anti-inflammatory effects in humans [127]. Plant extracts are classified as AChEI inhibitors (>50% inhibition), moderate inhibitors (30–50% inhibition), and weak inhibitors (30% inhibition) based on their level of potency [124]. Red algae extract from *H. musciformis* (7.21%) and *Pterocladia capillacea* (5.38%) had a minimal impact, while *Ochtodes secundiramea* extracts exhibited a moderate potency (48.59%) [128]. Antipyretics lower the increased body temperature. For example, flavonoids like baicalin, showed an antipyretic impact by inhibiting tumor necrosis factor (TNF) and preventing arachidonic acid peroxidation, which decreased the prostaglandin ratio and lessened fever and discomfort [129]. In comparison to normal paracetamol, the methanolic extract of *H. musciformis* and *Gracilaria dura* had a stronger antipyretic effect on albino mice and reduced body temperature for up to 4 h after administration [129].

#### 2.2.4. Anticoagulant

Galactans from red seaweed may serve as an alternate source for brand-new anticoagulant medications [130]. Seaweed has an anticoagulant property that may be related to the composition, molecular weight, sulfate concentration, and location of its polysaccharides, such as uronic acids, which carry a negative charge and have the ability to bind calcium ions, preventing the development of a clot [131]. The direct influence of algae on thrombin and the enhancement of antithrombin III may be the cause of the algal anticoagulation mechanism. Algal polysaccharides also prolonged crucial pathway-dependent coagulating durations, reduced platelet aggregation, and delayed activated partial thromboplastin time (APTT), suggesting the obstruction of intrinsic factors. Galactans from red seaweed are frequently potential alternate sources of intriguing anticoagulant medications [132]. Heparin-like anticoagulant effectiveness was demonstrated by sulfated galactans from *Grateloupia indica* [133]. The -O-SO3H group found in the red species carrageenans is crucial in preventing blood clots by blocking platelet aggregation [131]. About one-fifth of the activity of heparin was produced by carrageenans. Due to its increased sulfate concentration, λ-carrageenan demonstrated improved anticoagulant ability compared to κ- -carrageenan [131]. The anticoagulant action was boosted by depolymerizing agars obtained from *Porphyra yezoensis* and *Gracilaria birdiae* with the use of ultrasound [131].

#### 2.2.5. Macroalgae in Skincare

Macroalgae metabolites also improved skin’s brightness, remineralization, hydration, and firmness while also reducing the appearance of redness and blemishes, as well as UV damage [134]. The seaweeds *Asparagopsis armata*, *Gelidium corneum*, and *Corallina officinalis* have extracts that can be used in skincare products such as creams, oils, soaps, masks, and lotions to restore skin elasticity, softness, and whitening/lightening effects [3]. Certain anti-aging creams contain an amino acid that was isolated from *A. armata.* Mycosporine from several Rhodophyta species serves as a photoprotective ingredient [135].

## 3. Advancements in Algal Research

**Figure 2:**
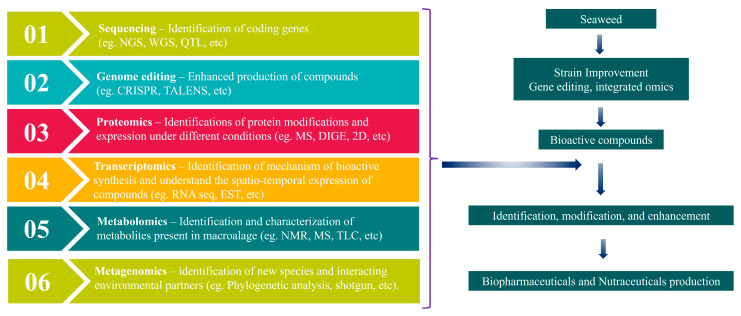
Omics approaches in macroalgal research.

Marine macroalgae are generally safe to consume and contain numerous bioactive compounds, but direct consumption alone is insufficient for therapeutic applications. Further formulation is necessary, considering digestibility and biochemical profiling, to develop algae-based drugs and food products [136]. However, conventional formulation methods, such as bioassays, can prove to be time-consuming and challenging. Fortunately, omics approaches have emerged as valuable tools for researchers seeking to manipulate and construct new algal metabolisms for use as potential therapeutic candidates [137]. Omics approaches are accelerating the development of algae-based therapeutics and food products (Figure 2). These approaches include genomics, proteomics, metabolomics, metagenomics, and other omics toolsets.

By comparing genomes across diverse macroalgae species, we gain insights into their unique adaptations, informing selection for cultivation. Transcriptomic and metabolic analyses pinpoint genes and metabolites responsible for valuable bioactive compounds like pigments, antioxidants, and polysaccharides, guiding strain selection and production optimization. CRISPR-Cas9 and gene silencing techniques enable the engineering of desirable traits like growth rate, stress tolerance, and compound production. Studying the macroalgal microbiome reveals its role in nutrient cycling, pathogen defense, and bioactive production, fostering beneficial interactions and improving cultivation practices. Genomics further guides the development of sustainable, closed-loop cultivation systems, maximizing resource efficiency and minimizing environmental impact. Omics technology, therefore, stands poised to revolutionize macroalgal research by unlocking their full potential for diverse applications.

### 3.1. Genomics

Genomics is a powerful omics technology that can reveal the metabolic and biosynthetic capabilities of organisms, enabling their manipulation for bioproduction and genetic engineering. Genomic tools not only help to identify the macroalgal compounds, but also help to characterize the identified bioactives [138].

#### 3.1.1. Genome Sequencing and Gene Mining

The number of available macroalgal genome sequences is still relatively insignificant compared to that of microalgae, due to the complexity of macroalgal genomes [139]. However, advanced next-generation sequencing technologies such as Hi-Seq, Mi-Seq, real-time sequencing, and pyro sequencing, have made whole-genome sequencing more efficient and accurate [140]. These technologies have been used to sequence a large number of marine macroalgae, with *Ectocarpus siliculosus* is the first marine macroalga that sequenced in 2010 [141]. By analyzing the genomic information, researchers can use gene mining to identify novel bioactive compounds with potential therapeutic or industrial applications [142]. For example, genomic analysis of the brown macroalga *Saccharina japonica* led to the discovery of a novel fucoxanthin synthase gene that can be used to produce fucoxanthin, a carotenoid with anti-cancer and anti-obesity properties [143].

#### 3.1.2. Genomic Analysis of Metabolic Pathways

Genomic analysis of macroalgae also provides insight into intracellular metabolic pathways, which are important for bioproduction [144]. For example, genomic analysis of the red macroalga *Chondrus crispus* has been used to identify genes involved in the production of carrageenan, a polysaccharide with a wide range of industrial applications [145].

#### 3.1.3. Web Resources for Genomic Analysis

In the era of genomics, a considerable number of web resources have been developed to support genomic analysis of marine macroalgae. These resources include:

- AlgaePath: A platform for predicting metabolic pathways in algae [146].
- pico-PLAZA: A database of genome information for algae [147].
- Joint Genome Institute (JGI) Portal: A portal that provides access to genome data and analysis tools from the JGI [148].
- Symbiodiniaceae and Algal Genomic Resource Database (SAGER): A database of genome data for Symbiodiniaceae algae [149].
- Organelle Genome Database for Algae (OGDA): A database of organelle genome data for algae [150].
- BioSyc: A database of metabolic pathways for algae [151].
- GOLD database: A database of genome data for over 8000 organisms (https://gold.jgi.doe.gov/ (accessed on 23 July 2023)).

However, an integrated database or web server specifically for macroalgae is yet to be developed.

#### 3.1.4. Genetic Engineering and DNA Barcoding

Genetic information from algae provides information on protein-coding genes, functional RNAs, enhancers, and regulatory elements [152]. This information can also be used for variety of applications, including:

- Phylogenetic analyses: To study the evolutionary relationships between distinct species of algae;
- DNA barcoding: To identify and classify algae species using short DNA sequences which are important for commercial and industrial applications. Genetic information can also be used for genetic engineering. Techniques such as RAPD, RFLP, hybridization, and AFLP are commonly used for barcoding [153]. Genetic engineering technologies such as TALENs, ZFNs, and CRISPR can also be used to alter the genetic material of macroalgae, enhancing the expression of genes with medicinal applications [137]. For example, CRISPR has been used to enhance the production of fucoxanthin in *Saccharina japonica* [154].

Nuclear genetic transformation has been established in the red microalgae *Cyanidioschyzon merolae* using homologous recombination technology, but these tools have yet to be used to alter macroalgal genomes [155]. Notably, recent efforts have seen improvements in the annotation of the giant kelp genome, aiming to establish it as a universal reference for genomic projects [156].

Overall, genomics is a powerful tool for marine macroalgae bioproduction and genetic engineering. By analyzing the genomic information, researchers can identify novel bioactive compounds, develop new bioproduction methods, and engineer macroalgae for improved traits. Also, genomics also acts as an important foundation through which macroalgal compounds can be utilized for industrial and biopharmaceutical purposes [157].

### 3.2. Transcriptomics

Transcriptomic profiling of macroalgae enables the identification of biochemical pathways involved in the production of medically significant compounds, aiding in the elucidation of these compounds [139]. For example, transcriptomic analyses revealed the response to blue light in *S. japonica*, revealing the overexpression of 7808 genes [158]. With advancements in technology, transcriptomic techniques have evolved from Expression Sequencing Tags (ESTs) to high-throughput methods, such as RNA-Sequencing and Biochips [159]. Transcriptomic studies focus on enhancing metabolites under specific growth conditions [160], and several transcriptome sequencing projects are available for this purpose. The Marine Microbial Eukaryotic Transcriptome Sequencing Project (MMETSP) contains more than 650 transcriptomes of diverse taxa [161], which can be used for transcriptomic studies.

In addition to identifying biochemical pathways, transcriptomic analysis can also identify the regulatory mechanisms involved in the accumulation of various metabolites [162]. For example, the characterization of *Laminaria digitata* identified the genes involved in alginate synthesis, while transcriptome analysis of *Ectocarpus siliculosus* revealed the upregulation of genes coding for chlorophyll a and c binding proteins under saline and oxidative stress conditions [163]. More recently, transcriptomic studies were utilized to identify the potential gene, to enhance the eicosapntemacnioc acid (EPA) production, which plays a key role in fluidity and organization of membrane [164]. Also, transcriptomic analysis can be helpful in identifying the differentially expressed compounds under varying environment conditions [165].

Overall, transcriptomic profiling is a powerful tool for marine macroalgae bioprospection. By analyzing the transcriptome data, researchers can identify genes and their expression associated with novel bioactive compounds, develop new bioproduction methods, and engineer macroalgae for improved traits.

### 3.3. Metagenomics

Metagenomics is a valuable tool for identifying algal strains and activating silent cryptic gene clusters involved in the synthesis of bioactive compounds [166]. Shotgun metagenomics is a high-throughput technique that enables the identification of multiple microbial communities [167]. However, studies on the algal microbiome and its associated community are still limited. Prokaryotes play a crucial role in enhancing the level of Vitamin B12 in algal species by strongly associating with the surface of macroalgae [168]. Metagenomic profiling of these prokaryotes identifies these organisms and helps to understand the interactions of macroalgae with other organisms. For example, metagenomic profiling has been used to identify the microbiome of *Porphyra* and *Pyropia* spp., which can enhance studies on the macroalgal microbiome [169]. Metagenomic analysis has also provided detailed information on the influence of the environment on bacterial communities [170].

Furthermore, Metagenomics is also a significant tool for bioprospecting, which can be employed for metabolic engineering of various algal strains. For example, metagenomic analysis of the microbiome of *L. digitata* revealed that algal polysaccharides provide a potential carbon source for bacteria, which can utilize them and produce numerous enzymes with biotechnological importance [171].

Overall, metagenomics is a powerful tool for marine macroalgae bioprospection. By analyzing the metagenome data, researchers can:

- Identify novel algal strains with potential for bioproduction of valuable compounds.
- Activate silent cryptic gene clusters in known algal strains to produce new bioactive compounds.
- Understand the interactions between macroalgae and their associated microbiome.
- Develop new bioprospection strategies for the discovery of novel enzymes and other biotechnologically important molecules.

### 3.4. Proteomics

Marine macroalgae exhibit high adaptability to their environment and produce various chemicals under different conditions [172,173]. Comparative proteomic analysis of macroalgae under varying environmental conditions can help identify differential protein expression within an algal system [174].

Several proteomic techniques can be employed to identify proteins in algal species, including:

- One-dimensional gel electrophoresis;
- Peptide fingerprinting;
- Sequencing;
- Two-dimensional electrophoresis;
- Mass spectrometry.

For example, proteomic analysis of the macroalga *Gracilaria changii* using two-dimensional electrophoresis and mass spectrometry has identified several novel proteins [175]. A deeper understanding of proteomics can aid in the redesign of the metabolic pathways of macroalgae, leading to increased production and accumulation of metabolites [176]. This can be used to develop new bioproduction methods for valuable compounds, such as drugs, nutraceuticals, and biofuels. However, proteomic studies on macroalgae, especially related to stress, are still limited compared to other plant species. One recent study used liquid chromatography-mass spectrometry (LC-MS/MS) and de novo sequencing to identify the increase in production of phycobiliproteins and superoxide dismutase in *Pyropis orbicularis* under desiccation conditions and the increase in phosphomannomutase and glyceraldehyde-3-phosphate in *Scytosiphon gracilis* in the presence of high concentrations of copper [177]. This study demonstrates the potential of proteomics to identify proteins that are involved in stress responses and other important metabolic pathways in macroalgae. Moreover, the combination of proteomic techniques including LC-MS, proteome analysis coupled with transcriptomic techniques can be used to evaluate and validate the potentials of macroalgal bioactive compounds [178].

Overall, proteomics is a powerful tool for marine macroalgae bioprospection. By analyzing the proteome data, researchers can:

- Identify novel proteins with potential for bioproduction of valuable compounds;
- Understand the molecular mechanisms of stress responses and other important metabolic pathways in macroalgae;
- Develop new bioprospection strategies for the discovery of new drugs, enzymes, and other biotechnologically important molecules.

### 3.5. Metabolomics

Metabolomics is the study of the complete set of metabolites produced by an organism. Marine macroalgae are known to produce a wide range of bioactive metabolites with potential applications in drug discovery, nutraceutics, and other industrial sectors. For example, macroalgae-derived metabolites have been shown to be effective against cancer [179], Alzheimer’s and other neurological disorders [180], and various infections [181]. Metabolomics is a valuable tool for assessing the quantity and quality of these metabolites in algae under natural and induced conditions since metabolites often vary based on environmental cues [182]. This information can be used to identify novel bioactive compounds with potential for therapeutic or industrial applications, as well as to develop new bioproduction methods for these compounds. Metabolomics plays a crucial role in drug discovery by profiling algal metabolites [181].

Various techniques are employed for metabolite analysis [183], including:

- Mass spectrometry (MS);
- Gas chromatography (GC);
- Nuclear magnetic resonance (NMR);
- Thin layer chromatography (TLC).

For example, NMR was used to perform microbial profiles of *L. digitata* and *Gracilaria conferta* [184], while GC-MS was used to quantify the levels of furanones in Delisea pulchra [185]. Metabolomic analyses have already led to the discovery of several new bioactive compounds from macroalgae [186]. For example, metabolic profiling of *Callophycus serratus* identified four bromophycolides that show activity against *Plasmodium falciparum* [187]. Additionally, metabolomics also helps to understand various metabolic pathways, such as defense response mechanisms in *Gracilaria vermiculophylla* [188]. Traditionally, metabolic engineering in macroalgae has relied on open-loop regulation, prioritizing specific biochemical pathways over system-wide efficiency [189]. This can lead to suboptimal production of desired metabolites. Closed-loop regulation offers a promising alternative, dynamically adjusting metabolic fluxes to maximize target com-pound yields [190]. Recent advancements in metabolic tools can empower researchers to implement closed-loop strategies, providing real-time feedback and optimized control over metabolic pathways.

Overall, metabolomics is a powerful tool for marine macroalgae bioprospection. By analyzing the metabolome data, researchers can:

- Identify novel bioactive compounds with potential for therapeutic or industrial applications;
- Develop new bioproduction methods for valuable compounds;
- Understand various metabolic pathways in macroalgae.

## 4. Macroalgal Industrial Extraction and Purification Methods

**Figure 3:**
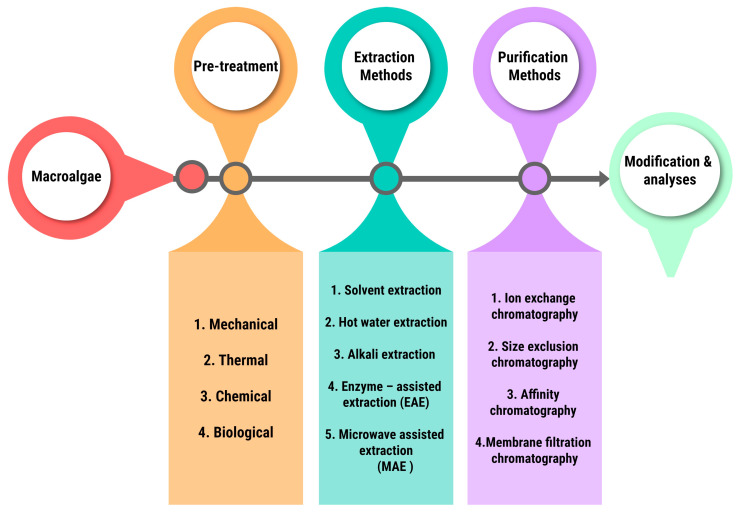
General industrial method of extraction and purification.

As from these resources there are many standardized procedures for the extraction and purification of the macroalgal components depending upon the industries. But the general method industrial approach is quite similar amongst all and is depicted in Figure 3.

The pretreatment methods change depending on the industry, for example the pretreatment method mostly followed for biochar production is thermal treatment, prominently pyrolysis. Macroalgal components can be extracted using a variety of methods, including solvent extraction and hot water extraction. The different advanced methods and the replaced ones are given below in Table 4. The operational principles techniques are briefly described in Table 5.

## 5. Future Prospectives

**Figure 4:**
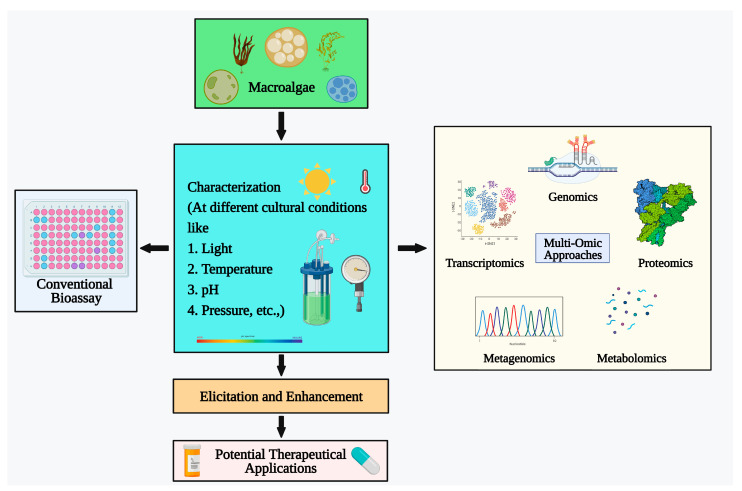
Macroalgal research in enhancing the level of bioactive compounds.

While the potential of macroalgae as oral drugs is undeniable, their complex cell walls pose a significant hurdle. Gastric enzymes struggle to break down these barriers, limiting the bioavailability of valuable bioactive compounds [136]. However, advancements in omics technologies offer promising solutions (Figure 4). The intersection of omics technology and macroalgae research holds immense potential for unlocking a treasure trove of biopharmaceuticals and nutraceuticals.

By harnessing the power of genomics, researchers can engineer new macroalgae strains with weaker cell walls or even replace them with readily digestible components. Additionally, omics-driven approaches can optimize the biochemical composition of macroalgae, enriching them with specific bioactive compounds. These breakthroughs would unlock the full potential of macroalgae as a sustainable and readily available source of oral drugs.

Omics will enable the identification of novel bioactive molecules in macroalgae with specific therapeutic properties, leading to the development of targeted drugs for diseases like cancer, diabetes, and neurodegenerative disorders. Moreover, transcriptomic and metabolomics analyses provide a deeper understanding of the biochemical pathways and metabolic signatures responsible for the biosynthesis of high-value compounds. This empowers researchers to optimize cultivation and extraction processes, maximizing the yield and purity of bioactive extracts while ensuring sustainable and cost-effective production. Beyond pharmaceuticals, omics technologies hold immense potential in revolutionizing the nutraceutical landscape. By identifying and optimizing macroalgae strains with enhanced levels of essential nutrients, such as vitamins, minerals, and omega-3 fatty acids, researchers can develop highly nutritious functional foods. Furthermore, proteomic and metagenomic analyses can unveil the functional properties of macroalgae, such as anti-oxidant and anti-inflammatory activities. This enables the creation of personalized functional food formulations tailored to individual dietary needs and preferences. Omics also plays a crucial role in safeguarding consumer health by providing valuable insights into the potential side effects and interactions of macroalgae-derived products. Toxicological studies guided by omics data can ensure the safety and efficacy of these novel nutraceuticals for widespread consumption.

Looking ahead, the integration of advanced artificial intelligence and machine learning in omics data analysis holds promise. This entails predicting promising macroalgae candidates and developing environmentally friendly cultivation practices to minimize the ecological impact.

In conclusion, the integration of omics technologies with macroalgae research presents a groundbreaking opportunity to unlock a treasure trove of biopharmaceuticals and nutraceuticals. By harnessing the power of these tools, we can not only revolutionize healthcare and nutrition but also contribute to a more sustainable future. The journey towards biovalorizing macroalgae is far from over, yet the potential rewards are immeasurable. As we continue to delve deeper into the secrets of the sea, we can expect even more exciting discoveries that will illuminate the path towards a healthier and more sustainable future for all.

## 6. Conclusions

In conclusion, macroalgae hold significant potential as an alternative source of food and medicine due to their high nutritional and therapeutic value. However, our current knowledge and research on seaweed are insufficient to fully exploit the vast potential of this natural resource. The application of advanced scientific technologies, particularly omics techniques, has begun to unravel the deeper potentials of macroalgae. With the integration of omics technologies, researchers have made notable progress in identifying new species, elucidating the abundance of bioactive molecules within macroalgae, enhancing adaptability and resistance to global warming and climate changes, developing novel compounds of greater significance, and establishing extensive databases for future research and development. Notably, macroalgae harbor a diverse array of pharmaceutical compounds that show promise in combating conditions such as cancer, inflammation, and neurological diseases. Further research should focus on scaling up seaweed production by optimizing culture conditions to meet the growing demand. It is crucial to thoroughly investigate the potential positive and harmful effects on the human body, as well as the economic significance of seaweed. Continued advancements in extraction techniques, purification, and fractionation of bioactive components will lead to the production of more effective and safe chemicals with antibacterial, antiviral, and anticancer properties.

While this review highlights some of the commonly studied macroalgae with nutraceutical and pharmaceutical potential, it is important to note that the marine ecosystem still harbors numerous unexplored organisms with diverse consumption and medicinal significance. Further exploration and research are necessary to unlock the full spectrum of possibilities offered by macroalgae and to contribute to the development of innovative and sustainable solutions for human health and well-being.

## Figures and Tables

**Table 1:** Comparison of Key Properties among Three Phyla of Macroalgae.

Properties	Brown Algae	Green Algae	Red Algae	Reference
Cell Wall Composition	cellulose, alginic acid, and fucoidan	Cellulose and pectin	cellulose, agar, and carrageenan	[23]
Reproduction	sexual and asexual	fragmentation, asexual spore formation, and sexual reproduction	sexual and asexual	[24]
Pigment	Fucoxanthin	chlorophyll	phycoerythrin and phycocyanin	[25]
Protein Content	<10%	10–20%	20–35%	[26]
Fatty Acid content	43.45%	43%	100%	[26]
EAA: NEAA *	0.73	0.72–0.97	0.98–1.02	[27]
Polysaccharides	fucoidans and laminarans	ulvan	carrageenan	[28]
Sulfated Polysaccharides	Fucans	heteropolysaccharides	galactans	[29]

* Essential Amino Acid (EAA) and Non-Essential Amino Acid (NEAA) ratio.

**Table 2:** List of nutrients present in macroalgae.

Nutrients	Organisms	Reference
Omega-3-Fatty acid	*Ulva* spp., *Acanthophora* spp., *Calliblepharis jubata*, *Macrocystis* spp., (Giant Kelp) *Gracilaria* spp., *Fucus distichus*, *Sargassum horneri, Porphyra crispata*, and *Undaria pinnatifida*	[26,35,36,37,38]
Vitamin C	*Porphyra ubmilicalis*, *Eisenia arborea*, *Gracilaria changii*, *Himanthalia elongate*, *Padilla tetrastomatica* and *Chaetomorpha alltellllilla*	[39,40,41,42]
Vitamin A	*Codium fragile*, *Padina pavonica*, *Gracilaria chilensis*, *Kappaphycus. striatum*, *Eucheuma denticulatum*, *Caullerpa lentillifera* and *Enteromorpha linza*	[43,44,45]
Vitamin E	*Alaria esculenta*, *Chondrus crispus*, *Furcellaria lumbricalis*, *Palmaria palmata*, *Ulva intestinalis*, *Ulva lactuca*	[36,46]
Mineral	*Laminaria digitata* (Oarweed), *Ecklonia cava* (Brown Algae), *Furcellaria lumbricalis*, and *Enteromorpha linza*, *Porphyra* (Nori),	[36,44,45,47,48]
Lutein	*Palmaria palmata*, *Chondrus crispus*,	[49,50]
Beta-Glucan	*Ulva lactuca*, *Fucus vesiculosus*, *Ascophyllum nodosum*	[51,52]
Calcium	*Fucus* spp., *Ascophyllum* spp., *Phymatolithon calcareum*, *Laminariales* (Kelp), *Hizikia fusiforme* (Hijiki), *Undaria pinnatifida*	[53,54]
Dietary Fiber	*Alaria esculenta* (Wakame), *Fucus serratus* (Serrated Wrack), *Chondrus crispus* (Irish Moss) and *Enteromorpha linza*	[44,45,47]
Polysaccharides	*Ulva fasciata*, *Ulva lactuca*, *Ulva reticulata*, *Codium* spp., *Enteromorpha* spp., *Dictyota dichotoma*	[55,56,57]
Chlorophyll	*Cladophora* sp., *Arthrospira* sp., *Enteromorpha*, *Ulva lactuca* and *Caulerpa* spp	[57,58,59]
Iodine	*Laminaria* spp. (Konbu) or (Kelp), *Sargassum muticum*, *Undaria pinnatifida*, *Chondrus crispus*, *Palmaria palmate*, *Saccharina* spp. and *Fucus vesiculosus*	[60,61,62]
Protein	*Alaria esculenta*, *Ulva* (Sea Lettuce), *Enteromorpha intestinalis*, *Palmaria palmata*, *Porphyra tenera* and *Vertebrata lanosa*, *Ascophyllum* (Rockweed).	[63,64,65]
Potassium	*Gracilaria* spp, *Palmaria palmate*, *Saccharina latissim*, *Laminaria digitata*	[66,67]
Carbohydrates	*Saccharina* spp. (Sugar Kelp), *Sargassum* spp. (Sargassum Weed), *Fucus* spp. (Bladderwrack), *Ascophyllum nodosum*, *Laminaria digitata*, *Undaria pinnatifida*	[47,68]
Antioxidants	*Cystophora monilifera*, *Cystophora pectinate*, *Zonaria angustata*, *Codium fragile*, *Cystophora pectinata*, *Ecklonia cava*, *Fucus vesiculosus*	[69,70]
Nitrogen	*Chaetomorpha maxima*, *Cladophora glomerata*, *Laminaria digitata* (Oarweed) and *Saccharina latissimi*	[66,71,72]
Phosphorous		[66]
Iron	*Desmarestia* spp., *Himantothallus* spp., *Saccharina japonica*, *Porphyra umbilicalis*, *Palmaria* spp., *Gracilariopsis* sp. *Sargassum* sp., *Ulva* sp.	[62,73]
Zinc	*Fucus vesiculosus*, *Fucus serratus*, *Laminaria digitata*, *Halydris siliquosa*, *Fucus spiralis*, *Pelvetia canaliculata*, *Corallina officinalis*	[74,75,76]

**Table 3:** Macroalgal Nutraceutical and Therapeutic applications.

Algal Ingredients	Biological Active Compound	Nutraceutical and Therapeutic Application	Reference(s)
Carbohydrate	Carrageenans	It can stimulate the immunological process by being an anti-inflammatory agent.	[77]
	Mannitol	Synthesization of drugs mainly for the diabetic patients, through which it would not increase the blood glucose level.	[78]
	Laminarin	Potential anticancer activity, prevents inflammation, immunomodulatory effects, used in cancer therapy and tissue engineering	[79,80]
	Cellulose	Cellulose nanofibers are used to improve the functional activities of drug delivery, tissue engineering and anti-microbial activity.	[81]
Lipids and fatty acids	Turbinaric acid	Possess anti-cancer, anti-bacterial, antioxidant activity as well as anti-inflammatory potential.	[82]
	Sphingolipids	Possess various biological activities including Antiviral, antitumor, immunostimulatory, neuritogenic activities	[83]
	Galactolipids	Acts as an antitumoral agents	[84]
	Palmitic acid	Used with ultrasounds to detect certain diseases and promotes the production of cosmetics as well as cleaning products as detergents.	[85]
Terpene	Diterpenes, Sesquiterpene	s. Strong cytotoxicity, inhibition of tumors and bacterial growth	[86]
Proteins	Lectins	The properties include antitumor, and antiviral.	[87]
	Phycobiliproteins	Natural coloring agents in chewing gums, dairy products and having immunomodulatory effects	[88]
	Ulvan Peptides	Important agent for immunomodulatory, antioxidant, anticancer activities	[89]
Amino acid	Mycosporine-like amino acid (MAA)	Have photoprotective properties as well as antioxidant, anti-cancer, wound healing, and skin protection applications.	[90]
	Taurine	Medical application to improve the heart and brain functions, and neuronal support.	[91]
Pigments	Phycoerythrobilin	To metabolize the metabolic channeling, food coloring agent, Therapeutic agent for Alzheimer’s disease, immunomodulatory and destroy the cancer cell proliferation activities	[92]
	Zeaxanthin	Main source of carotenoids, various health promoting properties such as antioxidant, anti-obesity, antitumor and hepatoprotective activity	[93]
	Caulerpin	Used as a kind of anti-inflammatory and anti-viral drug.	[94]
	Fucoxanthin	Can treat the acquired diseases like obesity, diabetes, cancer, and others.	[95]
Peptides	Wakame peptide jelly	For maintaining the food quality through antioxidant and anti-microbial activity.	[96]
	Hydrolysate	Application as a food and feed additive which can alter the protein properties and possess the antioxidant, anti-diabetic, cardioprotective and anti-microbial characteristics.	[97]
Secondary metabolites	Mahorone	Proposed application with food preservation or in cosmetic industries.	[98]
	Dimethylsulfoniopropionate (DMSP)	Main potentiality of neurotrophic or neuroprotective activity.	[99]
	Diphlorethol	The compound diphlorethohydroxycarmalol suppresses skin ageing.	[100]
	Quercetin	For the better functioning of heart and cancer. Also possess anti-inflammatory and antihistamine properties.	[101]
	Coumarin	The identified coumarins have anti-thrombic, anti-inflammatory, and vasodilatory activities. Among these warfarins are the identified best oral coagulant and a pesticide.	[102]
	Triphlorethol A	Have sleep promoting effects	[103]

**Table 4:** Advanced methods for conventional methods.

Method	Conventional method	Advanced method	Reference(s)
Pretreatment			
Mechanical methods	Solvents washing	-	
	Milling	High pressure homogenization Compressional puffing Hydrothermal process	[191]
	Drying		
2. Thermal methods	Conventional pyrolysis	Catalytic pyrolysis Hydrothermal carbonization Co-pyrolysis Microwave pyrolysis	[191]
3. Chemical methods	Solvent pretreatment	Ionic liquid	[192]
	Alkali pre-treatment	Chemo-sonic pretreatment with alkali treatment	
	Acid pretreatment	Thermo-acidic pretreatment (By replacing HCl with flue gas condensate)	
4. Biological methods	External enzyme	Mechano-enzymatic destruction by Haliatase	[191]
	In-situ enzymes	Saccharification	
Extraction methods			
Extraction	Solvent extraction Hot water extraction Alkali and acid extraction	Ultrasound-assisted extraction (UAE) Microwave-assisted extraction (MAE) Enzyme-assisted extraction (EAE)	[55]

**Table 5:** Operational Principle of different techniques.

Method	Technique	Operational Principle	Reference(s)
Mechanical method	High pressure homogenization (HPH)	HPH application upon the concentrated algal sledges, which is efficient for rupturing the tough cell wall. Hence this way reduces the homogenizing steps.	[192,193]
	Compressional puffing	Increase the yields of extracts using water and produced extracts of highest activities like [3] anti-bacterial, antioxidant etc.	[194]
	Hydrothermal process	Production of high value components from aquatic biomass with application of high temperature (180–250 ºC) and high pressure (20–40 bar)	[195]
Thermal methods	Catalytic process	The mechanism is poorly understood but the main use is in the oil industry to produce bio-oils by the combined processes of pyrolysis and catalytic cracking in the partial presence of oxygen.	[196]
	Hydrothermal carbonization	The production of hydrochar from wet biomass, and other organic biomasses as garbage, waste etc. by applying heat around 180–300 °C and pressure.	[197]
	Co-pyrolysis	The anoxygenic environment upgrades the produced oil with the characteristics of reduced water level, high calorific value and also to control the production cost for the same.	[198]
	Microwave pyrolysis	The standardized application of microwave at low temperature upon the sludge or the aquatic biomass with microwave absorbents increases the yield of syngas, pyrolysis oil, and biochar	[199]
Chemical methods	Ionic liquid	This method contributes to the lipid separation by the alteration of cell walls by ions from ionic solution.	[200]
	Chemo-sonic pre-treatment using alkali	Weakening of cell walls with application of alkali (Na^+^ and OH^−^) or surfactants leading to enhanced enzymatic hydrolysis for cellulose.	[201]
	Thermo-acidic pre-treatment	The lignocellulosic biomass is subjected to enzymatic hydrolysis for the fermentation of sugars.	[202]
Biological methods	Mechano-enzymatic destruction by Haliatase	A solvent free method which is effective cellulose and chitin degradation for fractionation of bioethnanol.	[203]
	Saccharification	Enzymatic conversion of cellulose and hemicellulose into high value compounds from waste resources hence reducing pollution.	[204]
Extraction	Ultrasound-assisted extraction (UAE)	Application of sound waves inducing pressure difference, and the waves converted into mechanical energy, which is capable to solubilize cell membrane, particle size reduction as well as enhanced interaction between targeted compounds and solvents.	[205]
	Microwave-assistedextraction (MAE)	Cell rupture through induced microwaves upon water and other polar molecules which causes the increase in intracellular temperature that can lead to evaporation of water and pressure on cell membrane.	[206]
	Enzyme-assisted extraction (EAE)	Development of phenolic compounds with high stability and high antioxidant activity by using water as the extraction solvent.	[207]

## Data Availability

Not applicable.
